# Evaluation of miR-9 and miR-143 expression in urine specimens of sulfur mustard exposed patients

**DOI:** 10.1515/intox-2015-0026

**Published:** 2015-12

**Authors:** Mostafa Khafaei, Shahram Samie, Seyed Javad Mowla, Akbar Ghorbani Alvanegh, Behnaz Mirzaei, Somaye Chavoshei, Ghamar Soltan Dorraj, Mostafa Esmailnejad, Mahmood Tavallaie, Mohammadreza Nourani

**Affiliations:** 1 Human Genetic Research Center, Baqiyatallah Medical Sciences University, Tehran, Iran; 2 Faculty of Biological Sciences, Department of Genetics, Tarbiat Modares University, Tehran, Iran; 3 Iranian Blood Transfusion Organization Research Center, Tehran, Iran; 4 System Biology Institute, Chemical injuries Research Center, Baqiyatallah Medical Sciences University, Tehran, Iran

**Keywords:** mustard, microRNA, bronchiolitis, apoptosis, inflammation, pathways

## Abstract

Sulfur mustard (SM) or mustard gas is a chemical alkylating agent that causes blisters in the skin (blister gas), burns the eyes and causes lung injury. Some major cellular pathways are involved in the damage caused by mustard gas such as NF-κb signaling, TGF-β signaling, WNT pathway, inflammation, DNA repair and apoptosis. MicroRNAs are non-coding small RNAs (19–25 nucleotides) that are involved in the regulation of gene expression and are found in two forms, extracellular and intracellular. Changes in the levels of extracellular microRNAs are directly associated with many diseases, it is thus common to study the level of extracellular microRNAs as a biomarker to determine the pathophysiologic status. In this study, 32 mustard gas injured patients and 32healthy subjects participated. Comparative evaluation of miR-9 and miR-143 expression in urine samples was performed by Real Time PCR and Graph Pad software. The Mann Whitney t-test analysis of data showed that the expression level of miR-143 and miR-9 had a significant decrease in sulfur mustard individuals with the respective p-value of 0.0480 and 0.0272 compared to normal samples, with an imbalance of several above mentioned pathways. It seems that reducing the expression level of these genes has a very important role in the pathogenicity of mustard gas injured patients.

## Introduction

Sulfur mustard is a chemical alkylating agent that was widely used in World War I (Adelipour *et al*., 2011). Sulfur mustard was also employed by Iraqi forces against Iranian civilians and soldiers between 1983 and 1988, resulting in extensive human casualties (Hefazi *et al*., 2005). Sulfur mustard causes blisters in the skin (blister gas), burns the eyes and causes lung injury (Balali-Mood & Hefazi, 2006, Vijayaraghavan, 1997). Since it has long-term debilitating effects and is fatal, it is considered a high-risk factor in chemical weapons (Smith *et al*., 1995). In broad terms: (1) mustard gas causes alkylation of proteins, membrane damage and glutathione (GSH) reduction, and (2) in target tissues (mainly the skin, eyes and respiratory system) it causes extensive necrosis, apoptosis, loss of tissue structure and acute and chronic inflammation (Balali-Mood & Hefazi, 2006; Hefazi & Balali-Mood, 2005). Much of the evidence suggests that oxidative stress or an imbalance between antioxidant enzymes and the products of oxidative reactions play a key role in the pathogenesis of the acute and chronic effects of exposure to mustard gas. The intracellular level of GSH has shown a significant correlation with the ability of mustard gas for alkylation (Papirmeister *et al*., 1985). Although the molecular and cellular basis for this pathology is not fully understood, some major cellular pathways are involved in the damage caused by mustard gas, such as NF-κB signaling, TGF-β signaling, WNT pathway, inflammation, DNA repair, apoptosis (Ruff & Dillman, 2007). The study of Gerecke *et al.* (2009) showed that the expression of more than 1000 microRNAs is increased following exposure to mustard gas. They are biologically classified as transcription factors, inflammatory factors, biosynthetic molecules and apoptosis inducers. No study has been done on the effects of mustard gas on microRNA expression (Gerecke *et al*., 2009). MicroRNAs are non-coding small RNAs (19–25 nucleotides) that are involved in the regulation of gene expression through binding to the three prime untranslated regions (3'UTR), so that the inhibition of gene expression is performed by both microRNA degradation and by preventing translation (Krol *et al*., 2004). It seems that if microRNA sequence is fully complementary to the microRNA in target 3'UTR, microRNA will be cut off, while if it is partially complementary, the inhibitory effect will be performed through inhibiting the translation (Khvorova *et al*., 2003). Since microRNAs play an important role in the regulation of gene expression, they have a direct relationship with the natural function of eukaryotic cells and thus any irregularity in their performance can cause a disorder and disease. Major microRNAs are intracellular but a number of them have recently been found extracellularly (in biologic liquids such as saliva, milk, serum, plasma and urine). Changes in the level of extracellular microRNAs are directly associated with many diseases, it is therefore common to study the level of extracellular microRNAs as a biomarker to determine the pathophysiological condition (Lin *et al*., 2005). Extracellular microRNAs employ specific methods to be protected from being cut off by nucleases such as packaging in exosomes and microvesicles, which causes the microRNAs to be resistant even in extreme conditions such as high temperature, variable PH and continuous freezing and thawing (Bartel, 2004). Exosomes are small vesicles with 30 to 100 nm diameter formed in the endosomes (Bergsmedh *et al*., 2001). Currently, the exosomes are considered important regulators in cell communication, with very different biological functions in the cell (Calin & Croce, 2006). There are approximately 500 microRNAs within an exosome. The microRNAs transferred through the exosome can modify the expression of many genes in the receptor cell (Deregibus *et al*., 2007). On the other hand, there is evidence stating that circulating exosomes can penetrate the membrane of renal glomerules, which underlies the presence of microRNAs in urine. Due to their high resistance, they are used as biomarker in order to determine the individuals’ pathophysiological condition (Chen *et al*., 2010). In this study, we measured the expression of miR-9 and miR-143 in urine samples obtained from sulfur mustard victims and compared it with normal controls. These two microRNAs are involved in many major pathways such as, TGF-β signaling, WNT pathway, NF-κB signaling, DNA repair and inflammation. The findings provide a novel insight into the epigenetic regulation of responses to sulfur mustard.

## Material and methods

### Ethics statement

The protocol was approved by the Medical Genetics Research Center of Noor and Research Center of Chemical Injuries, Baqiyatallah University of Medical Sciences. Consent form was signed by all healthy and diseased individuals before participating in the study.

### Study population

Thirty-two patients who had been injured by mustard gas before more than thirty years and who suffered from chronic respiratory, skin and eye effects were selected along with 32 healthy subjects. All participants in this study, which is a case-control study, were men with an average age of 45–52 years. All patients had documents approved by the military health services, moreover, these patients had manifestations of bronchiolitis obliterans (BOS) approved by high-resolution computerized tomography (HRCT). Lung scan data showed more than 25% expiratory air trapping. In addition, lung biopsy was obtained from sulfur mustard injured individuals and the results showed that they have been injured by mustard gas. Healthy subjects participating in this study were considered the control group. Their health was confirmed by the results of clinical experiments and chest X-ray film. Individuals with a history of chronic lung disease such as asthma, COPD, and lung cancer or autoimmune diseases (*e.g.,* connective tissue disorder, Graves’ disease) were excluded from the study and the normal individuals (control) who were selected had no history of smoking, drug or chemical poisoning.

### Sample collection

Samples were taken from the participants in the morning and kept at 4 °C. The samples were centrifuged for 15 minutes at 3 000 rpm, the supernatants were then stored in RNAase free tubes at –20 °C for the next steps.

### Total RNA and microRNA extraction

RNA extraction was performed using RiboEX-LS total RNA solution (GeneAll Biotechnology, Seoul, Korea). First, 750 μL of RiboEX-LS was added to 250 μL of urine supernatant, the mixture was then incubated for 10 min at 15 °C, then 200 μL of chloroform was added followed by shaking for 15 seconds and incubation at 15 °C. The mixture was then centrifuged for 15 minutes at 12 000 rpm. The supernatant was transferred into RNAase free tube and isopropanol (Merck, Darmstadt, Germany) was added at the same volume as the supernatant and incubated for 20 minutes at 15 °C, then centrifuged for 10 minutes at 12 000 rpm. The supernatant was discarded and 1 ml 75% ethanol (prepared in DEPC-treated water) was added and centrifuged for 5 minutes at 7 500 rpm, then the supernatant was discarded and the sediment was dried. Finally, the dried RNA was resolved using 40 ml of DEPC water-treated water. The micro tube containing RNA was stored at –80 °C. All procedures should be done under a chemical hood.

The RNA concentration and purity were confirmed by the spectrophotometric ratio using absorbance measurements at wavelengths of 260 nm and 280 nm on a Nanodrop 2000 (Thermo, Wilmington, USA), and isolated RNA was also analyzed by 2% agarose electrophoresis. Subsequently, the quantity assay for both samples was performed by Real-time-PCR machine (Applied Biosystem/Life Technologies, Grand Island, NY, U.S.A.)

### DNase I treatment

Generally DNase I treatment using DNase I kit(Takara, Dalian, China)is required to remove genomic DNA, not removed during RNA extraction, to prevent further problems. Although, DNA amplification is prevented using specially designed primers for cDNA synthesis for miRNA, this step will increase precision.

### Poly-A synthesis, cDNA synthesis and Real Time PCR

**Figure 1:**
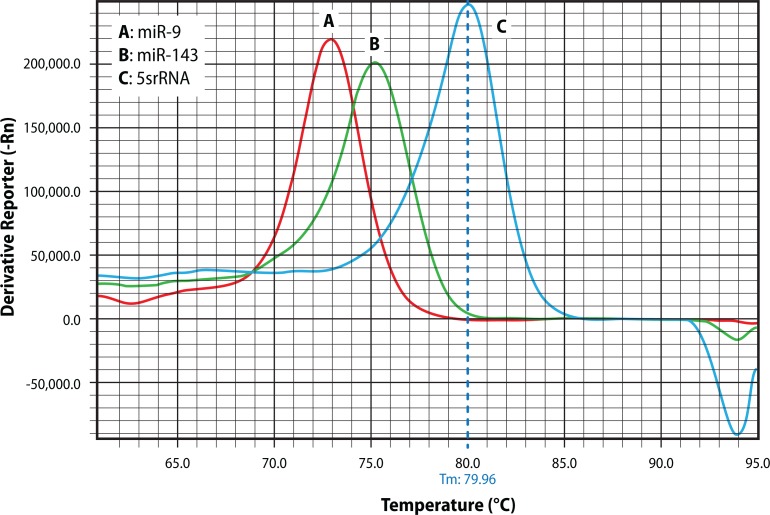
Post-amplification melting-curve analysis is a straightforward way to check real-time PCR reactions for primer-dirtier artifacts, presence of nonspecific products and to ensure reaction specificity A, B, C (miR-9, miR-143, 5srRNA, respectively) yields only one peak resulting from the specific amplification product.

Total extracted RNAs were converted into cDNA using poly (A) polymerase (Takara, Dalian, China) and poly adenylated at 37 °C for 1hour. cDNA synthesis was performed using specific primers and reverse transcriptase enzyme (Takara, Dalian, China). Human small nuclear, 5sRNA was amplified as an internal control to normalize the miRNA expression. Quantitative Real Time PCR analysis was performed using 2×SYBR Green PCR Master Mix (PEApplied Biosystem) and thermocycling program was of 40 cycles of 95 °C for 15 s and 60 °C for 1 min with an initial cycle of 95 °C for 15 min. The specificity of the reaction was confirmed by melt curve analysis (Figure 1).

### Statistical analysis

All the results were defined as mean ± standard deviation of expression level of miR-9 and miR-143 and Mann-Whitney t-test was used to compare gene expression level between control group and patients. Statistical analysis was performed by using GraphPad Prism and a p-value of <0.05 was considered statistically significant.

## Results

The study included 32 sulfur mustard injured patients and 32 healthy subjects as the control group. All participants in the study were men and mean± standard deviation of age of control and patients in the study was 3.39 and 4.66 years, respectively.

### Selection of internal control

According to the individuals’ physiological state, extracellular microRNAs are not circulating in an equal unit ratio, therefore a standard should be applied to normalize the data to reduce the impact of this factor in order to eliminate the systemic errors and changes that may naturally occur in any test, through which the real difference between the expression of microRNAs was realized in different samples The choice of an internal control to normalize the data is very important. The selection varies based on the type of sample, conditions or treatments used in different tests. In this study, 5SrRNA gene was selected as an internal control.

### Comparison of the expression of miR-9 and miR-143 in sulfur mustard patients and healthy individuals

**Figure 2:**
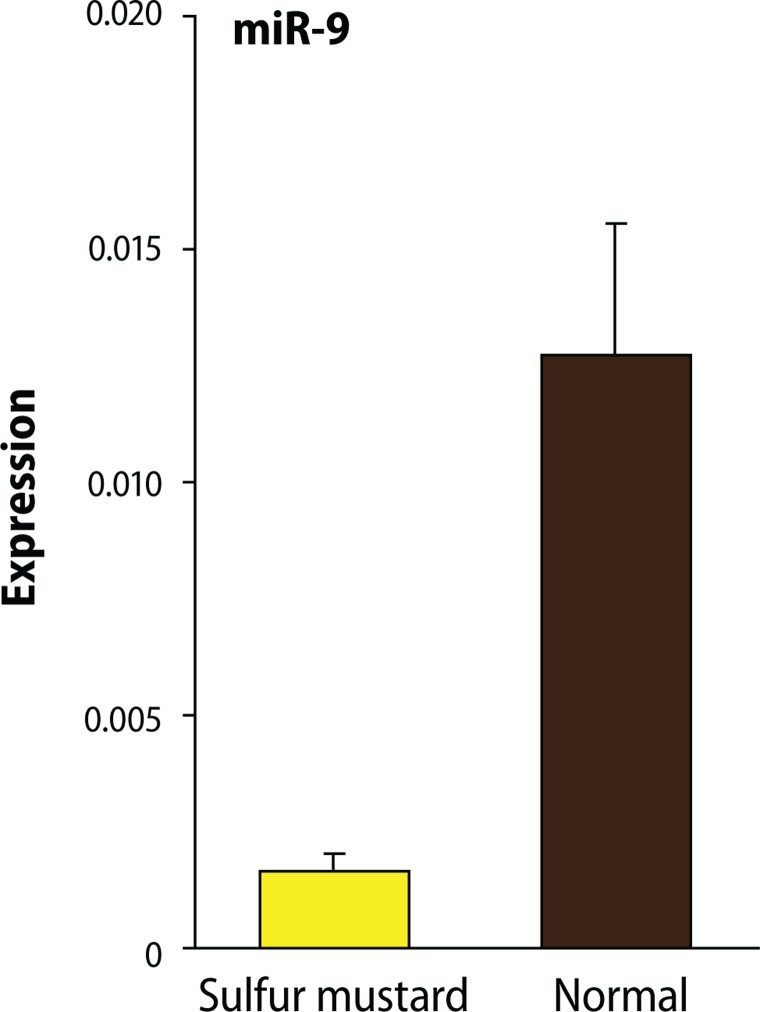
Decreases in the gene expressions of miR-9 in sulfur mustard patients comparing to healthy individuals. The expression Analysis showed that the expression level of miR-9 in injured individuals using Mann Whitney t-test analysis method with the *p*-value=0.0272 had a significant decrease compared to normal samples.

**Figure 3:**
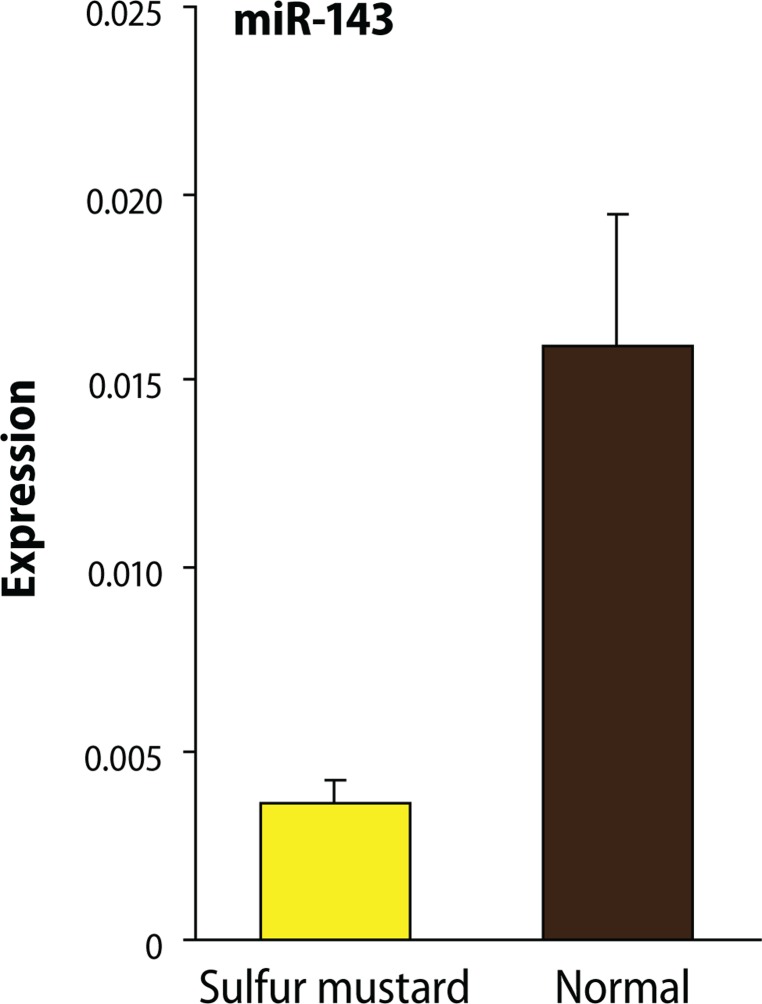
The expression Analysis showed that the expression level of miR-143 in injured individuals using Mann Whitney t-test analysis method with the *p*-value=0.0480 had a significant decrease compared to normal samples.

The expression analysis using Mann Whitney t-test showed that, compared to samples from normal subjects, in injured individuals the expression level of miR-143 and miR-9 exhibited a significant decrease with the respective p-value of 0.0480 and 0.0272 (Figures 2 and 3).

## Discussion

Mustard gas exposure damages different organs of the body, especially the skin, respiratory tract and eyes, which are affected through alkylation of cellular components such as RNA, DNA and it leads to damage on genetic and metabolic levels (Ludlum *et al*., 1984). The sulfur mustard delayed mechanism may be assumed to involve epigenetic perturbations linked to microRNA dysregulation. Although the majority of microRNAs are intracellular, a number of extracellular microRNAs have been recently discovered in the body's biological fluids such as serum, plasma, saliva, urine and milk (Lin *et al*., 2005). The secretory microRNAs are resistant and measurable in biological fluids and the collection of these liquids is non-invasive, changes in the spectrum of these extracellular microRNAs in the circulation have been correlated to different pathophysiological conditions (Chen *et al*., 2010). Thus the pattern expressed by extracellular microRNAs is a precise biomarker to determine the pathophysiologic status. According to the importance of the recent regulators (microRNAs), this study examined two circulating microRNAs, *i.e.* miR-9 and miR-143, in urine samples of sulfur mustard injured patients and healthy individuals. These two microRNAs were chosen because bioinformatics databases such as Mirpath and bioinformatics softwares such as PicTar, DIANA-microT-3.0 and Target Scan showed that both microRNAs (miR-9, miR-143) were involved in the regulation of important pathways such as MAPK signaling pathway, T cell receptor signaling pathway, TGF-β signaling pathway, cell communication, calcium signaling pathway, B cell receptor signaling pathway, cell cycle, Wnt signaling pathways, p53, VEGF signaling pathway, and some other pathways. TGF-β is one of the most important factors in the pathogenesis of mustard gas. Many studies have demonstrated that TGF-β was upregulated in the airway fibroblasts of SM exposed patients in comparison with control samples (Zhang *et al*., 2009). By reducing the expression of these microRNAs (miR-9, miR-143), a reduced expression of TGF-β in sulfur mustard injured patients is expected and in addition TGF-β is considered a moderating and suppressing factor in the inflammation, especially responsible for airway remodeling in mustard lung (Khaheshi *et al*., 2011). A comprehensive study conducted by Nourani and colleagues was aimed to determine the role of TGF-β and the severity of inflammation in sulfur mustard injured patients. TGF-β and its intracellular messenger molecules (SmadS) were found to have a special role in the modulation of inflammation, moderating and suppressing the fire provoked by other inflammatory cytokines and chemokines (Adelipour *et al*., 2011). Matrix metalloproteinases (MMPs) play an important role in the inflammatory process caused by mustard gas. Other studies have shown that TGF-β has an inhibitory role against MMPs in the inflammatory process (Kulkarni *et al*., 1993). According to the regulation of TGF-β signaling pathway by miR-9 and miR-143, the decreased level of expression of both microRNAs in sulfur mustard injured patients resulted in a lack of proper regulation of TGF-β pathway, and moreover, skin and lung complications were sustained (Long & Miano, 2011). Mustard gas can produce a cascade of free radicals which lead to the activation of inflammatory pathways through some transcription factors such as NF-κB (Ruff & Dillman, 2010). The sulfur mustard injured population often shows signs of pulmonary inflammation, usually bronchiolitis obliterans (BO), which results in tissue status change or tissue remodeling (Saber *et al*., 2012). The inflammation is caused by the imbalance between oxidants and antioxidants, in favor of the oxidants. Exposure of bronchial cells to the oxidant agents (ROS / RNS) leads to the expression of transcription factors such as NF-κB (Dahl *et al*., 1991). After arriving to the nucleus, this transcription factor binds to the agreed sequence in the upstream of inflammatory cytokine genes and causes them to be expressed (Tornatore *et al*., 2012). Similarly does this transcription factor lead to the expression of ICAM-1 gene, launching inflammatory and immunological interactions such as leukocytes passing through the blood barrier towards the lung. The increased expression of this gene causes a permanent presence of leukocytes in the lung and the secretion of inflammatory cytokines for a long time (Roebuck, 1999). Thus an increment expressing NF-κB may be caused by the decrease of the expression level of miR-9. Additionally, the NF-κB pathway leads to expression of IL-8 and IL-1 (Bazzoni *et al*., 2009, Yang *et al*., 2005). Further, these cytokines are negatively controlled by miR-9. Reduced expression of miR-9, increased expression of NF-κB and thus subsequently high production of IL-8 and IL-1 are expected in sulfur mustard injured individuals, which has been confirmed by several studies (Kehe *et al*., 2009). AP-1 complex subunits are c-fos and jun, controlled by miR-143 and miR-9, respectively. In a study Nourani and colleagues found that the expression of c-fos in airway epithelium increased compared to controls (Nourani & Yazdani, 2011). They further reported reduced expression of miR-9 and miR-143 leading to increased expression level of transcription factor mentioned above. Finally, the inflammatory pathways were activated in sulfur mustard injured patients as suggested also by several studies. Nourani *et al*. (2011) demonstrated a higher amount of MMP-9 and MMP-8 within the tears of sulfur mustard injured patients in comparison with healthy subjects (Shohrati *et al*., 2014). However, increase in the expression level of these MMPs leads to the production of VEGF (vascular endothelial growth factor) (Shiomi *et al*., 2005), which is also under control of miR-143 and miR-9. VEGF is an important component in lung restructuring. This factor leads to proliferation of new blood vessels, increased blood flow and edema. Extensive efforts have been made to control this protein. Anti-VEGF antibodies are prepared and currently applied. Sulfur mustard injured individuals and people with COPD and asthma show high levels of VEGF expression. Increase in MMP9 and VEGF expression leads to major problems in the eyes of sulfur mustard injured people and eventually causes corneal angiogenesis and destruction of the corneal stroma. Thus dry eye and angiogenesis are two important factors in the corneal damage of sulfur mustard injured people (Karami *et al*., 2011). Mustard gas is not only a genotoxic agent, but it also changes epigenetic processes. Epigenetic modification is one of the long-term and chronic impacts caused by mustard gas (Korkmaz *et al*., 2008). The epigenetic modification can be inherited from one generation to the next. The regulation of gene expression by epigenetic modification may be accomplished by two main mechanisms: changes in DNA methylation and histone modification. Many enzymes play a role in this process, including histone deacetylases (HDACs), histone acetyl transferases (HATs) and DNA methyl transferases (DNMTs). Korkmaz and colleagues concluded that mustard gas can activate the HDAC enzyme, which leads to the silencing of many beneficial genes such as genes encoding antioxidants and anti-inflammatory proteins. It is not quite clear how mustard gas leads to the activation of HDAC enzyme (Korkmaz *et al*., 2010). But this enzyme is under the control of miR-9, and decrease in the expression level of miR-9 is associated with an increased activity of HDAC. We conclude that, along with histopathology and preclinical findings, reduced expression levels of these genes in urine are promising biomarkers in patients exposed to SM.
